# Determinants of device success after transcatheter aortic valve replacement in patients with type-0 bicuspid aortic stenosis

**DOI:** 10.3389/fcvm.2023.1279687

**Published:** 2023-11-03

**Authors:** Zhicheng Xiao, Jing Yao, Xinmin Liu, Fei Yuan, Yunfeng Yan, Taiyang Luo, Moyang Wang, Hongliang Zhang, Faxin Ren, Guangyuan Song

**Affiliations:** ^1^ Department of Cardiology, Qindao University Medical College Affiliated Yantai Yuhuangding Hospital, Yantai, China; ^2^ Interventional Center of Valvular Heart Disease, Beijing Anzhen Hospital, Capital Medical University, Beijing, China; ^3^ Department of Cardiology, Fuwai Hospital, National Center for Cardiovascular Disease, Chinese Academy of Medical Science and Peking Union Medical College, Beijing, China

**Keywords:** transcatheter aortic valve replacement, bicuspid aortic valve, bulky calcification, device success, aortic stenosis

## Abstract

**Background:**

Clinical evidence of transcatheter aortic valve replacement in patients with type-0 bicuspid aortic valve was relatively scarce.

**Aims:**

Our goal was to explore determinants of device success after transcatheter aortic valve replacement in patients with type-0 bicuspid aortic valve morphology.

**Methods:**

In this retrospective multicenter analysis, we included 59 patients with symptomatic severe aortic stenosis with type-0 bicuspid aortic valve morphology who underwent transcatheter aortic valve replacement. Type-0 bicuspid aortic valve was identified with multidetector computed tomography scans. The technical success rate was 89.8%, and the device success rate was 81.4%. Patients were divided into a device success group and a device failure group according to Valve Academic Research Consortium- 3 criteria.

**Results:**

When we compared the two groups, we found that the ellipticity index of the aortic root and the presence of bulky calcifications at the commissure were statistically different (ellipticity index 35.7 ± 1.7 vs. 29.7 ± 1.1, *p* = 0.018; bulky calcification at the commissure, 54.5% vs. 4.5%, *p* < 0.001). Further multivariate logistic analysis showed that bulky calcification at the commissure had a negative correlation with device success (odds ratio 0.030, 95% confidence interval 0.003–0.285, *p* = 0.002). Yet there was no statistical correlation between the ellipticity index and device success (odds ratio 0.818, 95% confidence interval 0.667–1.003, *p* = 0.053).

**Conclusions:**

The presence of bulky calcifications at the commissure is negatively correlated with device success after transcatheter aortic valve replacement in patients with type-0 bicuspid aortic valve.

## Introduction

Twenty-one years have passed since Dr. Alain Cribier performed the first transcatheter aortic valve replacement (TAVR) (1). TAVR has been shown to be an effective and safe treatment for low-to-high surgical risk patients with symptomatic aortic stenosis (AS) compared with surgical aortic valve replacement (2–4). Bicuspid aortic valve (BAV) occurs in 1%–2% of the population and accounts for 22%–28% of patients over 80 years old with AS who need surgery. Due to their special anatomy, patients with BAV were excluded from previous randomized controlled trials (5). In 2017, Yoon et al. found BAV patients after TAVR had a similar 2-year mortality, lower procedural success, and a higher rate of paravalvular regurgitation (PVR) compared with patients with a tricuspid aortic valve (6). Forrest et al. found that the 1-year unadjusted risk of mortality was lower in BAV patients after TAVR (7), demonstrating that TAVR was effective and safe in BAV patients.

Type-0 BAV is an aortic valve (AV) morphology with only two equal cusps and two symmetric sinuses, absent any raphe or fusion between the leaflets. This classification of BAV was first introduced by Sievers through observation of 304 surgical specimens (8). Using the Sievers classification, two special phenotypes can be divided according to the direction of the cusps within the short axis of the heart plane, the laterolateral (side-to-side) or anteroposterior (front-and-back) BAV (9). Thus far, several classification methods for BAVs have been suggested but none of them have shown a correlation with clinical outcomes (9). Moreover, there is a paucity of data concerning type-0 BAV morphology, which is less frequently seen in Western populations. We sought to explore the determinants of device success after TAVR in BAV anatomy, with a particular focus on type-0 BAV morphology.

## Methods

### Study design and patients' selection

Consecutive patients with BAV with symptomatic AS from two Beijing centers, Fuwai Hospital and Anzhen Hospital, were included in the study between November 2020 and April 2022. All patients were diagnosed with severe AS by echocardiography if they met one of the following criteria: mean transvalvular pressure gradient (PGmean) of AV ≥40 mmHg; peak transvalvular velocity (Vmax) of AV ≥4 m/s, or AV area ≤1.0 cm^2^. Fifty-nine patients were recognized as type-0 BAV through multidetector computed tomography scanning. All data were analyzed by the core laboratory, and therapeutic strategies were discussed by the cardiac multidisciplinary team before the operation. The therapeutic strategies were based on ESC/EACTS Guidelines and ACC/AHA Guidelines for valvular heart diseases. For patients who needed CABG or surgical intervention, they were mostly recommended and accepted cardiac surgery, except for patients who were judged moderate to severe frailty. For symptomatic patients with severe AS who were 65–80 years of age and had no anatomic contraindication to transfemoral TAVR, either SAVR or transfemoral TAVR was recommended after shared decision-making. For patients who were >80 years of age or for younger patients with a life expectancy <10 years and no anatomic contraindication to transfemoral TAVR, transfemoral TAVR was recommended in preference to SAVR. The study was conducted in accordance with the Code of Ethics of the World Medical Association (Declaration of Helsinki) for experiments involving humans.

### TAVR procedures

The procedures were performed as described previously (10). All procedures were performed with the patients under local or general anesthesia, with intubation or laryngeal mask airway, in the hybrid catheterization laboratory. The means of inducing anesthesia was determined by the anesthetist, based on the patient's general condition and pulmonary function. Transfemoral access was the first choice when the femoral artery was of adequate caliber. Four types of prosthetic aortic valves were used for TAVR (Supplementary Figure S1), including the Venus-A (Venus Medtech, Inc. Hangzhou, China), Vitaflow (MicroPort Scientific Corporation, Shanghai, China), TaurusOne (Peijia Medical Co, Suzhou, China), and SAPIEN 3 (Edwards Lifesciences, Irvine, CA, USA). About half the self-expanding valves were implanted with the new generation of retrievable delivery systems.

### Study end points

The end points of the study were defined according to Valve Academic Research Consortium- 3 criteria (11). Periprocedural mortality was defined as death occurring ≤30 days after the index procedure or >30 days but during the index hospitalization. Device success met all the criteria at 30 days: technical success; freedom from death; freedom from surgery or intervention related to the device or to a major vascular or access-related or cardiac structural complication; intended performance of the valve (mean gradient <20 mmHg, peak velocity <3 m/s, Doppler velocity index ≥0.25, and less than moderate aortic regurgitation).

### Special notes

Calcification of the leaflet free edge referred to obvious calcification along the edge, over 2/3 the length of it. Bulky calcification at the commissure was determined by visual assessment using multidetector computed tomography transverse planes and maximum intensity projections. The ellipticity index of the aortic root was calculated as (long axis/short axis-1) × 100%, on the plane 5 mm above the annulus. The oversizing ratio was calculated as (prosthesis nominal perimeter/multislice computed tomography-derived annular perimeter-1) × 100%. A pacemaker was implanted if a high-degree of or complete atrioventricular blocking occurred and lasted for more than 24 h after the operation. Two special phenotypes of type-0 BAV were divided according to the direction of the cusps within the short axis of the heart plane, the laterolateral (side-to-side) or anteroposterior (front-and-back) BAV. Laterolateral indicates direction of the cusps is approximately parallel to the direction of the atrial septum within the short axis of the heart plane. Anteroposterior means cusps arranged in a front-and-back direction, with the orifice approximately perpendicular to the atrial septum.

### Statistics

Continuous variables were described as mean ± standard deviation and compared using the unpaired Student t-test, in case they matched normal distribution. Categorical variables were described as numbers and percentages and were analyzed with the *χ*^2^ test or the Fisher exact test. Variables with *p*-values < 0.1 on univariate analysis were entered into multivariate logistic regression models. All statistical analyses were performed using SPSS version 20.0 (IBM Inc., Armonk, NY, USA) with two-tailed significance set at 0.05.

## Results

### Baseline characteristics

Fifty-nine patients were included in the study; 25 of them (42.4%) were male. The mean Society of Thoracic Surgeons predicted risk of mortality score was 4.3 ± 1.7%. The PGmean of the AV before the operation was 62.7 ± 21.9 mmHg; the ejection fraction was 52.6 ± 15.4%. There were 17 patients (28.9%) who had moderate or severe mitral regurgitation, and 9 patients (15.3%) with moderate or severe tricuspid regurgitation. Most type-0 BAVs had an oval annulus with an ellipticity index of 30.8 ± 7.6%. Calcification of unilateral or bilateral leaflet free edges was found in up to 24 patients (40.7%). The ascending aorta was dilated in most patients, with an average diameter of 44.1 ± 7.0 mm at its widest plane. Two patients had ascending aorta diameters over 55 mm, but they were too fragile to undergo cardiac surgery, and we performed TAVR as a compromise formula. Nine patients' ascending aorta diameters ranged from 50 mm to 54 mm, without additional risk factors or coarctation. Thirteen patients had ascending aorta diameters from 45 mm to 49 mm, and the other patients had diameters less than 45 mm. In addition, the coronary ostial height of type-0 BAV patients was relatively high. The average left coronary ostial height was 15.5 ± 3.4 mm, and the right coronary ostial height was 16.9 ± 3.9 mm (Table 1).

**Table 1:** Baseline characteristics.

*N* = 59	
Male	25 (42.4%)
Age, year	69.3 ± 7.0
NYHA grading	
1	3 (5.1%)
2	16 (27.1%)
3	36 (61%)
4	4 (6.8%)
STS predicted risk of mortality score, %	4.3 ± 1.7
COPD	2 (3.4%)
DM	14 (23.7%)
HP	29 (49.2)
Cr level, μmol/L	84.4 ± 23.5
Prior PCI	3 (5.1%)
Prior CABG	0
Prior cardiac surgery	1 (1.7%)
Peripheral artery disease	3 (5.1%)
Prior stroke/TIA	2 (3.4%)
Atrial fibrillation	10 (16.9%)
Ejection fraction, %	52.6 ± 15.4
PGmean of AV, mmHg	62.7 ± 21.9
Vmax of AV, m/s	4.8 ± 1.0
LVEDD, mm	49.0 ± 7.5
Aortic regurgitation	4 (6.8%)
Mitral regurgitation	17 (28.9%)
Tricuspid regurgitation	9 (15.3%)
Laterolateral direction	47 (79.7%)
Calcification score, mm^3^	664.4 ± 561.8
Bulky calcification at commissure	8 (13.6%)
Calcification of free edge	
None	35 (59.3%)
Unilateral	21 (35.6%)
Bilateral	3 (5.1%)
Maximum diameter, mm	37.0 ± 5.9
Minimum diameter, mm	25.5 ± 4.4
Ellipticity index, %	30.8 ± 7.6
Annulus diameter, mm	24.4 ± 3.1
LVOT diameter, mm	25.2 ± 5.0
STJ diameter, mm	33.0 ± 5.0
Ascending aorta diameter, mm	44.1 ± 7.0
Angle of heart, degree	53.4 ± 11.3
Height of LCA, mm	15.5 ± 3.4
Height of RCA, mm	16.9 ± 3.9

AV, aortic valve; CABG, coronary artery bypass grafting; COPD, chronic obstructive pulmonary disease; Cr, creatinine; DM, diabetes mellitus; HP, hypertension; LCA, left coronary artery; LVEDD, left ventricular end diastolic diameter; LVOT, left ventricular outflow tract; NYHA, New York Heart Association; PCI, percutaneous coronary intervention; PG, pressure gradient; RCA, right coronary artery; STJ, sinotubular junction; STS, Society of Thoracic Surgeons; TIA, transient ischemic attack.

### Operative procedures and outcomes

In total, 96.6% of the patients were operated on via a transfemoral access. Two patients underwent TAVR via transcarotid and transaxillary accesses, respectively, since transfemoral access was not feasible. Only 1 patient had vascular complications. Based on the annular perimeters, calcification distribution and restriction above the annulus, we usually chose the “downsize strategy”, with a 2.3 ± 8.1% oversizing ratio. Two patients died during the perioperative period, including 1 who had a valve-in-valve implantation because of paravalvular regurgitation and died of delayed occlusion of the left coronary artery after the operation. The other patient was discharged from the hospital in stable condition, and the cause of death was unknown. Valve-in-valve procedures were performed in 6 patients (10.2%) due to moderate to severe PVR after deployment of the first valve. Four patients (6.8%) had permanent pacemaker implants after the operation. The technical success rate was 89.8%, and the device success rate was 81.4%. Moderate-to-severe mitral regurgitation or tricuspid regurgitation was significantly reduced postoperatively (28.9% vs. 13.6%, 15.3% vs. 5.1%) (Table 2).

**Table 2:** Procedures and outcomes.

*N* = 59	
General anesthesia	24 (40.7%)
Femoral artery access	57 (96.6%)
Predilation	58 (98.3%)
Type of prosthetic valves	
Venus-A	27 (45.8%)
Vitaflow	22 (37.3%)
TaurusOne	7 (11.9%)
SAPIEN 3	3 (5.1%)
Size of prosthetic valves, mm	
23	24 (40.7%)
24	12 (20.3%)
26	9 (15.3%)
27	8 (13.6%)
29	3 (5.1%)
30	3 (5.1%)
Oversizing ratio, %	2.3 ± 8.1
Retrivable delivery system	28 (47.5%)
Vascular complications	1 (1.7%)
Structural complications of heart	0
Valve-in-valve	6 (10.2%)
Surgery	0
Permanent pacemaker	4 (6.8%)
Stroke	0
Technical success	53 (89.8%)
Device success	48 (81.4%)
Perioperative death	2 (3.4%)
Ejection fraction (post-TAVR), %	53.4 ± 12.0
PGmean of AV (post-TAVR), mmHg	16.5 ± 10.8
Vmax of AV (post-TAVR), m/s	2.2 ± 0.6
PVR (post-TAVR)	
1	20 (33.9%)
2	3 (5.1%)
3	1 (1.7%)
Mitral regurgitation (post-TAVR)	8 (13.6%)
Tricuspid regurgitation (post-TAVR)	3 (5.1%)

AV, aortic valve; PG, pressure gradient; PVR, paravalvular regurgitation.

### Determinants of device success

Patients with device failure or device success were divided into 2 groups. When we compared the 2 groups, we found no significant differences in the STS score, medical history, ejection fraction, or PGmean of the aortic valve. The orientation of the aortic cusps also did not affect the device success rate. There was no statistical difference in unilateral or bilateral free edge calcification and overall calcification scores between the 2 groups. And we found no difference between early generation devices (unretrievable delivering systems) and retrievable delivering systems. It's worth pointing out 4 patients out of the 31 patients who used the unretrievable delivering systems underwent a second valve implantation. Two patients out of the 28 patients who used the retrievable delivering systems underwent a second valve implantation. However, bulky calcification at the commissure was found in 54.5% of the patients in the device failure group and in 4.5% in the device success group (Table 3), implying that bulky calcification had an inverse relationship with device success. In addition, the morphology of the aortic root was more elliptical in the device failure group than in the device success group (ellipticity 35.7 ± 1.7% vs. 29.7 ± 1.1%, *p* = 0.018) (Table 3).

**Table 3:** Characteristics of patients with device success.

	Device failure, *n* = 11	Device success, *n* = 48	*P* value
Male	6 (54.5%)	19 (39.6%)	0.37
Age, year	66.9 ± 2.3	69.9 ± 1.0	0.21
NYHA grading			0.56
1	0	3 (6.3%)	
2	4 (36.4%)	12 (25%)	
3	7 (63.6%)	29 (60.4%)	
4	0	4 (8.3%)	
STS predicted risk of mortality score, %	4.3 ± 0.4	4.3 ± 0.3	0.90
COPD	1 (9.1%)	1 (2.1%)	0.34
DM	3 (27.3%)	11 (22.9%)	1
HP	3 (27.3%)	26 (54.2%)	0.11
Cr level, μmol/L	76.7 ± 5.7	86.2 ± 3.5	0.23
Prior PCI	1 (9.1%)	2 (4.2%)	1
Peripheral artery disease	1 (9.1%)	2 (4.2%)	1
Prior stroke/TIA	0	2 (4.2%)	1
Atrial fibrillation	3 (27.3%)	7 (14.6%)	0.57
Ejection fraction, %	54.4 ± 5.3	52.1 ± 2.2	0.66
PGmean of AV, mmHg	64.3 ± 9.2	62.4 ± 2.9	0.8
Vmax of AV, M/s	5.0 ± 0.4	4.8 ± 0.1	0.7
LVEDD, mm	49.2 ± 2.7	49.0 ± 1.1	0.94
Aortic regurgitation			0.59
No	5 (45.5%)	22 (45.8%)	
Mild	6 (54.5%)	22 (45.8%)	
Moderate	0	4 (8.3%)	
Laterolateral direction	9 (81.8%)	38 (79.2%)	1
Calcification score, mm^3^	825.6 ± 159.8	626.0 ± 88.5	0.32
Bulky calcification at commissure	6 (54.5%)	2 (4.5%)	<0.001
Calcification of free edge			0.51
None	8 (72.7%)	27 (56.3%)	
Unilateral	3 (27.3%)	18 (37.5%)	
Bilateral	0	3 (6.3%)	
Maximum diameter, mm	38.6 ± 1.0	36.6 ± 0.9	0.32
Minimum diameter, mm	24.9 ± 0.8	25.6 ± 0.7	0.61
Ellipticity index, %	35.7 ± 1.7	29.7 ± 1.1	0.018
Annulus diameter, mm	25.5 ± 0.9	24.2 ± 0.4	0.21
LVOT diameter, mm	26.7 ± 1.1	24.8 ± 0.7	0.26
STJ diameter, mm	32.9 ± 1.1	33.0 ± 0.8	0.94
Ascending aorta diameter, mm	43.2 ± 1.2	44.3 ± 1.1	0.67
Angle of heart, degree	53.1 ± 1.6	53.4 ± 1.6	0.93
Height of LCA, mm	16.0 ± 1.2	15.4 ± 0.5	0.61
Height of RCA, mm	17.8 ± 1.0	16.7 ± 0.6	0.42
General anesthesia	5 (45.5%)	19 (39.6%)	0.99
Type of prosthetic valves			0.77
Venus-A	5 (45.5%)	22 (45.8%)	
Vitaflow	4 (36.4%)	18 (37.5%)	
TaurusOne	2 (18.2%)	5 (10.4%)	
SAPIEN 3	0	3 (6.3%)	
Size of prosthetic valves, mm			0.29
23	3 (27.3%)	21 (43.8%)	
24	2 (18.2%)	10 (20.8%)	
26	4 (36.4%)	5 (10.4%)	
27	2 (18.2%)	6 (12.5%)	
29	0	3 (6.3%)	
30	0	3 (6.3%)	
Oversizing ratio	−1.5 ± 2.9	3.2 ± 1.1	0.079
Retrievable delivery system	6 (54.5%)	22 (45.8%)	0.60

AV, aortic valve; COPD, chronic obstructive pulmonary disease; Cr, creatinine; DM, diabetes mellitus; HP, hypertension; LCA, left coronary artery; LVEDD, left ventricular end diastolic diameter; LVOT, left ventricular outflow tract; NYHA, New York Heart Association; PCI, percutaneous coronary intervention; PG, pressure gradient; RCA, right coronary artery; STJ, sinotubular junction; TIA, transient ischemic attack.

**Figure 1:**
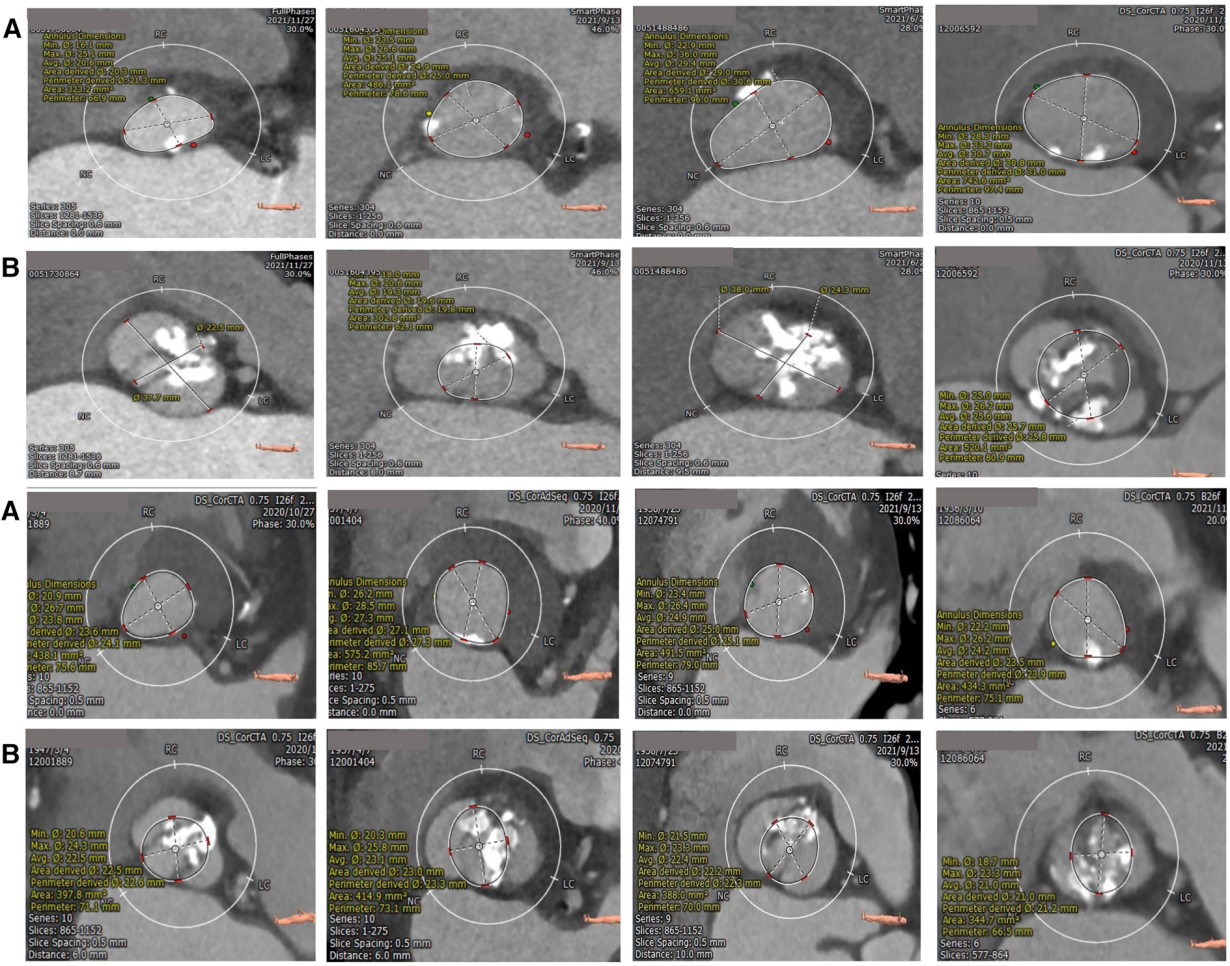
Calcification distribution of annulus (**A**) and bulky calcification at the commissure (**B**) superior and inferior are the same patients. The bulk calcification appeared primarily on the plane 6–8 mm above the annulus.

Univariate logistic regression analysis, including age, STS score, and other factors, showed that bulky calcification at the commissure and the ellipticity index of the aortic root correlated with device success. Variables with *p*-values < 0.1 on univariate analysis were entered into multivariate logistic regression models. Multivariate logistic regression analysis, including bulky calcification at the commissure, the ellipticity index and oversizing ratio as covariates, showed that bulky calcification at the commissure negatively correlated with device success (odds ratio, 0.030, 95% confidence interval 0.003–0.285, *p* = 0.002) (Table 4). A total of 8 patients had bulky calcification at the commissure, with calcification mostly observed 6–8 mm above the annulus (Figure 1).

**Table 4:** Independent correlates with device success.

	Univariate logistic		Multivariate logistic	
	OR (95% CI)	*p*-value	OR (95% CI)	*p*-value
Male	0.55 (0.15–2.04)	0.369		
Age	1.07 (0.96–1.18)	0.211		
STS predicted risk of mortality score	1.03 (0.69–1.53)	0.895		
Atrial fibrillation	0.455 (0.097–2.146)	0.320		
Ejection fraction	0.99 (0.95–1.04)	0.655		
LVEDD	0.997 (0.91–1.09)	0.942		
Laterolateral direction	0.844 (0.157–4.545)	0.844		
Calcification score	0.999 (0.998–1.001)	0.325		
Bulky calcification at commissure	0.036 (0.006–0.230)	<0.001	0.030 (0.003–0.285)	0.002
Calcification of free edge	2.138 (0.570–8.019)	0.260		** **
Aortic regurgitation	1.243 (0.415–3.728)	0.697		
Ellipticity index	0.835 (0.720–0.967)	0.016	0.818 (0.667–1.003)	0.053
Annulus diameter	0.876 (0.712–1.076)	0.206		
LVOT diameter	0.922 (0.802–1.061)	0.258		
Angle of heart	1.003 (0.945–1.063)	0.931		
Oversizing ratio	1.080 (0.989–1.178)	0.086	1.028 (0.911–1.160)	0.657
Retrievable delivery system	0.705 (0.189–2.628)	0.603		

CI, confidence interval; LVEDD, left ventricular end diastolic diameter; LVOT, left ventricular outflow tract; OR, odds ratio; STS, Society of Thoracic Surgeons.

## Discussion

TAVR in bicuspid AS has been an important focus of research in recent years. Due to the lack of evidence in BAV patients who have undergone TAVR in early large randomized controlled trials, the long-term prognosis of these patients is still under discussion. Sievers' classification can describe the morphology of the aortic valve but has poor clinical prognostic value. The BAV anatomical spectrum classification was developed by Michelena et al., who hoped to provide predictive value for clinical outcomes (9). Due to the complexity of its classification system, its clinical application is limited. The correlation with clinical outcomes needs to be further verified. Yoon et al. found that a calcified raphe and excess leaflet calcification were associated with increased risk of procedural complications and 2-year all-cause mortality (12). This research is the first to focus on the impact of calcification degree and calcification distribution of leaflets on clinical outcomes. Calcified raphe mainly exists in type-1 BAVs, yet for type-0 BAVs with no calcified raphe or excess leaflet calcification, which factors influence device success rate or prognosis of the patients is not well answered. Ielasi et al. compared type-0 BAV and type-1 BAV patients and observed a higher rate of mean transprosthetic gradient ≥20 mmHg in patients with type-0 BAV postoperatively (13). Compared with type-1 BAVs, type-0 BAVs are more likely to affect the prosthetic valve's well dilation. However, the study found no statistical difference of Valve Academic Research Consortium-2 device success rate between the two groups. Based on previous studies, we are interested in patients with type-0 bicuspid aortic stenosis, and wonder whether TAVR in these populations will differ from others.

Ascending aorta dilatation was common in our study. In 2022 ACC/AHA Aortic Disease Guideline (14), it is recommended that in patients with a BAV, a diameter of the aortic root or ascending aorta of 5.0 cm–5.4 cm, and an additional risk factor for aortic dissection, surgery to replace the aortic root, ascending aorta, or both is reasonable, when performed by experienced surgeons in a Multidisciplinary Aortic Team. And in our study, patients with a diameter of the aortic root or ascending aorta of 5.0 cm–5.4 cm, were excluded from additional risk factors, such as aortic growth rate 0.3 cm/year, aortic coarctation or “root phenotype” aortopathy. So they don't meet the criteria of class 2a indication. The two patients with a diameter of the ascending aorta of >5.5 cm, who had an STS mortality risk score of >8, were too fragile to undergo SAVR and ascending aorta replacement, and eventually chose TAVR as a compromise formula. The therapeutic strategies were discussed by the cardiac multidisciplinary team before the final decision. BAV aortopathy can be divided into three dilatation phenotypes: ascending phenotype (70%), root phenotype (20%) and extended phenotype. And right-left fusion phenotype of BAV is thought associated with aortic root dilation (9). Whether type-0 BAV is associated with one of the dilatation phenotypes is still unknown. Most patients in our study had ascending aorta dilatation, but not aortic root dilatation. In fact, of the 11 patients with a diameter of the ascending aorta >5.0 cm, only one patient didn't achieve device success because of a second valve implantation. In Lei et al.'s study, long diameter of the sinus of valsalva is 37.1 ± 4.2 mm and short diameter is 26.7 ± 3.2 mm. Data of ascending aorta aren't shown in their study (15). In Yoon et al.'s study, the proportion of ascending aorta dilatation (≥5.0 cm) is 2.2%, smaller compared with our study (12). And ascending aorta-major diameter of type-0 BAV is 36.6 ± 4.0 mm, as shown in Ielasi et al.'s paper (13).

The device success rate was 81.4% in our study. Six patients needed a second valve implantation during the operation and one patient suffered from major vascular complication, leading to a technical success rate of 89.6%. Two patients had peak velocity of over 3 m/s 1 month after the operation, two patients had severe paravalvular leakage, and two patients died of sudden death within 1 month. It's worth pointing out that one of the patients who died and one of the patients with severe paravalvular leakage had undergone second valve implantation. The device success rates ranged from 83.4% to 96.5% in previous studies (6, 7, 13). Yoon et al. compared procedural and clinical outcomes in TAVR for bicuspid vs. tricuspid AS, and found TAVR in bicuspid AS had similar prognosis with tricuspid AS, but lower device success rate (6). Type-1 BAV was the major type in this study, and type-0 BAV accounted for only 12.8%. The proportion of self-expanding valves accounted for 34.4%, and balloon-expanding valves were used in more than half the patients. Furtherly they found lower device success rates in bicuspid AS mainly appeared when using early generation devices, not suitable for new generation devices. Forrest et al. also compared outcomes in patients with bicuspid vs. tricuspid AS undergoing TAVR. The device success rate for type-0 BAV in the study was 96.5% and all-cause mortality was 1.7%, better results compared with previous studies (7). This might be partly due to improvements in devices and partly due to increased proficiency. Compared with their papers, our study showed similar all-cause mortality, but lower device success rate. Reasons for lower device success rate might be as follows: First, the study groups differed. We focused on patients of type-0 BAV, which accounted for about 10% of total BAV patients, and these patients had a trend toward a lower device success rate and a higher rate of mean trans prosthetic gradient ≥ 20mmHg, compared with type-1 BAV; Second, selecting patients of improper anatomy might be another reason. Third, using early generation unretrievable delivering system might lead to inappropriate placement of valves and increase the risk of a second valve implantation.

How to select a prosthetic valve of proper size is hard for patients of BAV. Annular-based sizing with minimal oversizing was thought accurate in BAV, meanwhile prosthesis under-expansion was common (16). Kim et al. compared annular vs. supra-annular sizing for TAVR in BAV patients. Supra-annular sizing might result in divergent size selection in approximately 40% of cases, with potential worsening in a large proportion of patients (17). Yet whether the discovery is fit for type-0 BAV needs further verification. In our paper, we also used annular-based sizing and the oversizing ratio was 2.3% ± 8.1%. Since annulus of most type-0 BAV is elliptical, we wonder supra-annular sizing based on intercommissure distance may not accurately reflect the structure.

Direction of cusps is an important parameter of type-0 BAV. Lei et al. assessed the procedural and clinical results of TAVR for nonraphe bicuspid aortic stenosis with coronary vs. mixed cusp fusion (15). Nonraphe BAV is similar to type-0 BAV, coronary and mixed cusp fusion morphology was analogous to anteroposterior and laterolateral classification. Thirty-day mortality was 7.0% in the study and had no relationship with cusp fusion morphology. Device success rates were not presented in the study, but they found need for a second valve was similar between the two groups. Our research demonstrated the direction of cusps was irrelevant with device success rates.

Type-0 BAV is different from type-1 BAV in many aspects. The absence of a raphe and 2 symmetric sinuses means that with TAVR in type-0 BAV, the prosthetic valve is rarely pushed to one sinus. In our study, the presence of bulky calcification was found to be an independent risk factor for device failure. The reasons may be as follows: The bulky calcification causes displacement or insufficient expansion of the prosthetic valve or poor adherence to the aortic wall, thus resulting in the use of a second valve, moderate-to-severe paravalvular regurgitation, or a high postoperative transvalvular pressure gradient. PVR is associated with an increased 5-year risk of mortality, and insufficient expansion of the prosthetic valve may impact device durability (18, 19). Probably due to the shear stress of the blood, we found that calcification was mainly present at the free edge of the leaflets, in a linear pattern. An elliptical aortic root may also lead to underexpansion of the prosthetic valve, thereby leading to the presence of paravalvular regurgitation and high transvalvular gradients. However, in multivariate logistic regression analysis, the elliptical index showed no statistical significance.

### Study limitations

This is a retrospective observational study with limited sample, so the results need further confirmation. Besides, the population we focused on are type-0 BAV patients, which would reduce the generalizability of the conclusions. We discussed determinants of device success in our paper, while determinants of other endpoints, such as all-cause mortality, were not explored.

## Conclusions

This is the first study focusing on patients with type-0 BAV who underwent TAVR. We found that bulky calcification at the commissure negatively correlated with device success. Our research provides a new aspect for cardiac interventionists, to evaluate the risk of the procedure in this special population. And retrievable delivering system should be considered once bulky calcification at the commissure is noticed. However, the sample size of this study is small and requires verification by larger samples.

## Data Availability

The original contributions presented in the study are included in the article/Supplementary Material, further inquiries can be directed to the corresponding author.
