# Supplementary figures and images for: Determinants of device success after transcatheter aortic valve replacement in patients with type-0 bicuspid aortic stenosis
Source: Front Cardiovasc Med. 2023 Nov 3;10:1279687. doi: 10.3389/fcvm.2023.1279687 (PMC10657190; doi:10.3389/fcvm.2023.1279687)

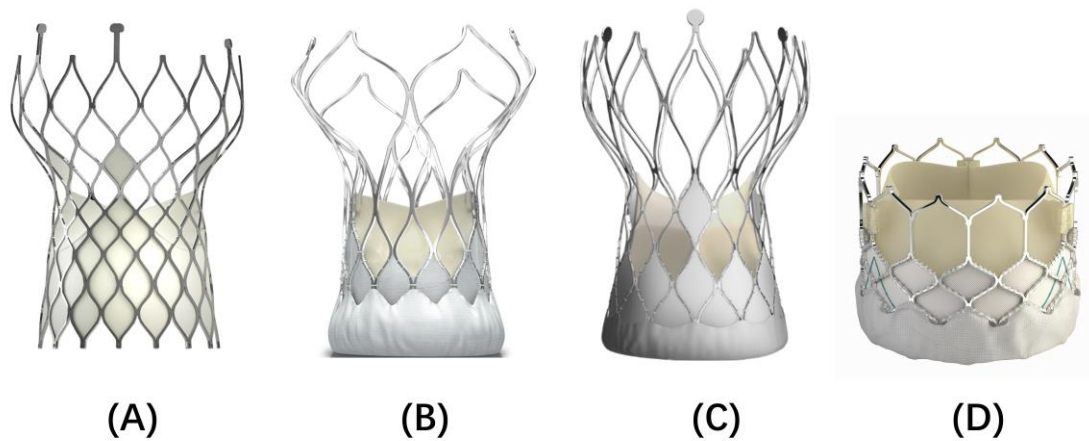

FIGURE S1

Prosthetic valves used: (A) Venus-A; (B) Vitaflow; (C) TaurusOne; (D) SAPIEN 3

Supplement: Supplementary file 1 [file Presentation1.pdf]
